# The Effect of Antidepressant Treatment on Neurocognitive Functions, Redox and Inflammatory Parameters in the Context of COVID-19

**DOI:** 10.3390/jcm12227049

**Published:** 2023-11-12

**Authors:** Eliza Samaryn, Beata Galińska-Skok, Aleksander Nobis, Daniel Zalewski, Mateusz Maciejczyk, Monika Gudowska-Sawczuk, Barbara Mroczko, Anna Zalewska, Napoleon Waszkiewicz

**Affiliations:** 1 Department of Psychiatry, Medical University of Bialystok, 15-272 Bialystok, Polanddaniel.zalewski@sd.umb.edu.pl (D.Z.);; 2 Department of Hygiene, Epidemiology, and Ergonomics, Medical University of Bialystok, 15-022 Bialystok, Poland; 3 Department of Biochemical Diagnostics, Medical University of Bialystok, 15-269 Bialystok, Poland; 4 Department of Neurodegeneration Diagnostics, Medical University of Bialystok, 15-269 Bialystok, Poland; 5 Department of Restorative Dentistry, Medical University of Bialystok, 15-276 Bialystok, Poland

**Keywords:** antidepressant treatment, antidepressants, COVID-19, SARS-CoV-2, SARS-CoV-2 antibodies, depression, oxidative stress, inflammation, neurocognitive functions, CRP, D-dimers

## Abstract

Inflammation is an important component of the etiopathology of depression that uses oxidative and nitrosative stress (O&NS) and elevated inflammatory markers. SARS-CoV-2 infection is also associated with abnormal inflammatory processes, which may impair effective treatment of depression in COVID-19 survivors. In the presented study, thirty-three hospitalized patients with major depressive disorder (MDD) were started on antidepressant treatment, and twenty-one were re-evaluated after 4–6 weeks. The control group consisted of thirty healthy volunteers. All participants underwent neuropsychiatric evaluation, biochemical blood and urine analyses. The results of the research demonstrated positive correlations of the Hamilton Depression Rating Scale (HAM-D) scores with serum catalase (CAT) and urinary S-Nitrosothiols levels, and the Beck Depression Inventory (BDI) scores with serum reduced glutathione (GSH) and superoxide dismutase (SOD) levels. Depressed patients with a history of COVID-19 prior to the treatment had higher urinary nitric oxide (NO) levels and lower serum glutathione peroxidase (GPx) levels. In the control group, COVID-19 survivors had higher levels of urinary N-formylkynurenine (NFK). Our results suggest that the antidepressant treatment has a modulating effect on O&NS, reduces depressive symptoms and improves cognitive functions The present study does not indicate that clinical response to antidepressant treatment is associated with COVID-19 history and baseline SARS-CoV-2 antibody levels. Nevertheless, further research in this area is needed to systematize antidepressant treatment in COVID-19 survivors.

## 1. Introduction

The global COVID-19 pandemic has affected society in multiple ways. In particular, it has impacted mental health through exposure to prolonged stress, anxiety and a sense of uncertainty [1,2]. It has been proven that stressful life events may cause vulnerability to depression [3]. Since the outbreak of the COVID-19 pandemic, there has been a significant increase in the prevalence of affective disorders including depression and anxiety alongside other mental disorders [4].

Currently, depressive disorder is the most common potentially life-threatening mental disorder, and a leading cause of disability [5]. Research indicates that an estimated 3.8% of the world population suffers from depression, including 5% of adults, roughly twice as many women as men [6]. The etiology of depression results from a complex interaction between genetic, social, psychological and biological factors [7]. The following factors are all indicated to have a significant impact on the development and severity of depression: dysregulation of the hypothalamic–pituitary–adrenal axis, neurotransmission disorders including serotonin, chronic inflammation with increased inflammatory cytokines, O&NS, mitochondrial dysfunction and decreased levels of brain-derived neurotrophic factor (BDNF) [8,9,10,11,12,13].

Available data indicate that SARS-CoV-2 infection can exacerbate the symptoms of depression and even stimulate the development of this disorder. It is related to the direct pathomechanism of SARS-CoV-2 infection and coronavirus neurotropism and the indirect immune response simultaneously. It can lead to a generalized inflammatory “cytokine storm”, blood–brain barrier damage, glial activation and consequent neuroinflammation with structural brain changes [14,15,16]. As a result of pro-inflammatory cytokines, the COVID-19 infection is accompanied by an increased activation of the kynurenine pathway and production of reactive oxygen species (ROS), which leads to oxidative damage [15,17]. A study conducted by Ahmed et al. demonstrated that O&NS contributes to the etiology and severity of COVID-19 infection. The levels of individual reactive oxygen and nitrogen species in serum, including peroxynitrite, were significantly higher in patients with COVID-19 than in healthy subjects [18]. In another study conducted on COVID-19 subjects with severe symptoms, postmortem examination revealed decreased levels of glutathione, which is the main antioxidant in all tissues. Reduced levels of this enzyme in neurons may lead to neuronal cell death [19]. In a meta-analysis, Ceban et al. showed that about one-third of individuals experienced persistent fatigue, and more than one-fifth of individuals suffered from cognitive impairment 12 or more weeks after COVID-19 diagnosis. Moreover, elevated proinflammatory markers, including elevated D-dimer levels, were also found in a subgroup of post-COVID individuals [20]. COVID-19 infection in depressed patients may exacerbate the body´s inflammation, leading to increased cognitive dysfunction and risk of developing neurodegenerative disorders [21,22].

There are studies that demonstrate several beneficial effects of antidepressants such as a reduction in inflammation, a reduction of pro-inflammatory cytokines and oxidative stress activity as well as improvements in neurocognitive functions [23,24,25,26]. Antidepressant treatment can attenuate the intensity of depression through the modulation of inflammatory pathways, brain structure transformation and synaptic plasticity [27]. It has been shown that many antidepressants reduce microglia activation and are effective in modulating the immune response [28,29]. There is growing preclinical and clinical evidence for the antioxidant effects of antidepressants. However, their mechanism of action has not been fully understood [30,31,32,33]. The research on animal models indicates that antidepressants reduce the markers of oxidative stress in the brain, the liver and peripheral tissues, and also modulate antioxidant barrier activity including SOD, CAT and GSH. The antioxidant effect has been shown to be dose-, length- and treatment-regimen-dependent [33]. Caruso et al. demonstrated that long-term treatment combined with the antidepressants fluoxetine or vortioxetine can prevent the oxidative stress associated with the depressive phenotype and memory impairment in a non-transgenic animal model of Alzheimer’s disease [34]. Moreover, vortioxetine has been developed to treat cognitive dysfunctions [35]. It has been observed that antidepressants, including selective serotonin reuptake inhibitors, can mitigate the cytokine storm in COVID-19 patients [36]. Studies indicate that the drugs mentioned above may constitute a promising adjunctive treatment for COVID-19 infection [37,38,39] and reduce the risk of death and hospitalization in COVID-19 patients [36]. Furthermore, antidepressants in COVID-19 survivors can positively affect their mood and improve cognitive functions [20]. Nevertheless, a chronic exposure to increased inflammation may impair or diminish the effectiveness of antidepressants. It is worth mentioning that patients with high inflammation demonstrated a poor response to conventional antidepressant therapies. Studies show that a comorbid immune background of inflammatory diseases is not only a risk factor for a depressive episode but is also considered a factor in drug resistance and recurrence of depression [40]. Therefore, the treatment of depression poses a new therapeutic challenge in the context of the COVID-19 pandemic.

The present study evaluated the effect of antidepressant treatment on the clinical and biochemical aspects of depression including changes in redox and inflammatory parameters. Moreover, another objective of the study was to examine the influence of the history of SARS-CoV-2 infection on the therapeutic effect of depression treatment, neurocognitive functions and analysis of selected inflammatory parameters. 

## 2. Materials and Methods

This study was conducted at the Department of Psychiatry of the Medical University of Bialystok. The study participants were recruited among the patients hospitalized in the General Psychiatric Wards of the Independent Public Psychiatric Health Care Center in Choroszcz and the Department of Psychiatry at the Medical University of Bialystok. The recruitment process began in December of 2021 and was completed in February of 2023. The study was approved by the Ethics Committee of the Medical University of Bialystok (permission: APK.002.281.2021) and was carried out in accordance with the Helsinki Declaration and the Guidelines for Good Clinical Practice.

### 2.1. Study Design and Participants

Each participant of the study was an adult Polish citizen of the Caucasian race and presented informed consent to participate in the study. The study and control groups were selected symmetrically in terms of age (18–65 years old) and gender. Exclusion criteria included pregnancy or breastfeeding, neurocognitive diseases, serious head trauma with a history of subsequent cognitive impairment, obesity (BMI > 30 kg/m^2^), steroid therapy, addiction to psychoactive substances including alcohol and active somatic comorbidity with a proven inflammatory basis. On the day of inclusion in the experiment, all study participants were physically and psychiatrically examined with the assessment of neurocognitive functions. Biological material was collected from them for analysis (the 1st measurement), and blood basic biochemical parameters were examined to assess the general health. The severity of stress associated with the COVID-19 pandemic was assessed by using a Polish version of the Impact of Event Scale—Revised (IES-R) and a self-administered questionnaire considering the respondent´s demographics. The Hamilton Depression Rating Scale (HAM-D), the Beck Depression Inventory (BDI) and the Hamilton Anxiety Rating Scale (HAM-A) were used to assess symptoms of depression and anxiety. A neuropsychological assessment was performed using the Verbal Fluency Test (VFT), WAIS-R Digit Span Test (DST), Trail Making Test (TMT) Parts A and B, Stroop Color Word Test (SCWT) and the California Verbal Learning Test (CVLT). In hospitalized patients with depression who were started on antidepressant treatment, the testing procedure (psychiatric examination with the assessment of neurocognitive functions and collection of biological material) was repeated after 4–6 weeks (the 2nd measurement).

The study group consisted of patients with a diagnosis of unipolar depression who qualified for hospitalization and treatment due to clinical deterioration. To exclude psychiatric comorbidities, the M.I.N.I. questionnaire was used (Mini International Neuropsychiatric Interview). The diagnosis of depression was performed according to ICD-11 and SCID-1 criteria and confirmed by an experienced psychiatrist (B.G.-S.). Initially, 37 participants were recruited into the study group; however, 4 of them had to be excluded from the study due to not meeting the inclusion and exclusion criteria (BMI > 30 kg/m^2^). Finally, 33 patients (women = 20, men = 13, mean age = 40.7) were included in the study group, among whom 15 participants confirmed a positive history of COVID-19 and a symptomatic course of the disease. The mean time between COVID-19 infection and study examination was 15 months. After conducting the verification by testing IgG anti-protein N and IgG anti-protein S-RBD antibodies to the SARS-CoV-2 virus, past contact with the virus was confirmed in 21 subjects. Participants subjectively rated the severity of SARS-CoV-2 infection on a scale of 1 to 10 points. In addition, they graded taste and olfactory impairment during COVID-19 using a three-point scale: 0 points—unchanged, 1 point—weakened, 2 points—loss, 3 points—altered.

Twenty-one patients, among them 13 women and 8 men, (including 15 with a confirmed history of SARS-CoV-2) were re-evaluated after a period of the antidepressant treatment with serotonergic transmitter modulating properties. The drug was chosen by the treating physician based on the clinical picture, previous response to treatment and possible side effects. The antidepressant treatment was initiated at the start of hospitalization and included selective serotonin reuptake inhibitors (SSRIs) (escitalopram *n* = 1, fluoxetine *n* = 1, sertraline *n* = 3), serotonin and norepinephrine reuptake inhibitors (SNRIs) (duloxetine *n* = 7, venlafaxine *n* = 3), amitriptyline (*n* = 1), vortioxetine (*n* = 1), mirtazapine (*n* = 1) and combination therapy of duloxetine + bupropion (*n* = 3). Drug dosages were used according to individual clinical response and tolerance to treatment. Escitalopram dosing was started at 5 mg per day and increased to a maximum of 10 mg per day. Fluoxetine dosing was started at 10 mg per day and increased to a maximum of 40 mg per day. Sertraline dosing was started at 25 mg per day and increased to a maximum of 100 mg per day. Duloxetine dosing was started at 30 mg and increased to a maximum of 90 mg per day. The dose of bupropion in combination treatment with duloxetine was up to a maximum of 300 mg per day. Venlafaxine dosing was started at 75 mg per day and increased to a maximum of 225 mg per day. Amitriptyline dosing was started at 25 mg and increased to a maximum of 150 mg per day. Mirtazapine dosing was started at 10 mg and increased to a maximum dose of 45 mg per day. Lastly, vortioxetine dosing was started at 5 mg and increased to a maximum dose of 10 mg per day (Table 1). The response to the antidepressant treatment was measured as improvements in scores on the HAM-D, BDI and HAM-A scales before and after the treatment.

The control group consisted of 30 healthy volunteers (women = 20, men = 10, mean age = 42.5) with no former history of psychiatric disorders who met the inclusion and exclusion criteria. The M.I.N.I. (Mini International Neuropsychiatric Interview) questionnaire was used to identify the control group. The mean time between COVID-19 infection and examination was 9.2 months. Among these individuals, 21 out of 30 confirmed a positive history of COVID-19, but after a verification of antibodies to SARS-CoV-2, contact with the virus was found in 23 study participants.

### 2.2. Blood and Urine Collection

The biological material tested consisted of 10 mL of blood and 10–15 mL of urine. Venous blood was collected from each fasting participant in the morning by qualified staff using sterile disposable equipment. In the next step, a fraction of the collected material was analyzed for basic biochemical parameters (complete blood count, potassium, sodium, creatinine, alanine transaminase, aspartate transaminase, C-reactive protein, thyroid-stimulating hormone, total cholesterol, low-density lipoprotein, high-density lipoprotein, triglycerides and D-dimers) using an MPW M-DIAGNOSTIC centrifuge and a Cobas Integra 400+ analyzer (Roche, Basel, Switzerland). 

Subsequently, the rest of the serum was frozen in Eppendorf tubes (Eppendorf, Germany, Hamburg) and stored at −80 °C for other assays including anti-SARS-CoV-2 antibodies (anti-N IgG and anti-S-RBD IgG) and redox parameters. 

Urine samples were collected from the midstream of the first-morning urine and centrifuged at 1300× *g* for 10 min at 4 °C. (MPW 351, MPW Med. Instruments, Warsaw, Poland). Subsequently, the supernatant was collected, frozen and stored in Eppendorf tubes at −80 °C until biochemical analysis was performed.

### 2.3. C-Reactive Protein and D-Dimer Assays

The method used to determine C-reactive protein (CRP) and D-dimer parameters was the immunoturbidimetric method.

### 2.4. SARS-CoV-2 Antibody Assays

IgG antibodies to the nucleocapsid protein (anti-N IgG) and the receptor-binding domain (RBD) of the S1 subunit of the spike protein (anti-S-RBD IgG) of SARS-CoV-2 were measured on an Alinity analyzer (Abbott, Chicago, IL, USA) according to the manufacturer’s guidelines using a chemiluminescent microparticle immunoassay (CMIA). The measurements for anti-N IgG antibodies, titers ≥ 1.4 and for anti-S-RBD IgG antibodies ≥ 50 AU/mL were positive.

### 2.5. Redox Assays

The parameters determined in serum and urine were the following: kynurenine (KN), N-formylkynurenine (NFK), dityrosine (DT), tryptophan (TRY), superoxide dismutase (SOD), catalase (CAT), glutathione peroxidase (GPx), reduced glutathione (GSH), 4-hydroxynonenal (4-HNE), nitric oxide (NO), S-Nitrosothiols and peroxynitrite.

All reagents for redox assays were purchased from the company Sigma-Aldrich (Nümbrecht, Germany/Saint Louis, MO, USA). Antioxidant enzymes were detected in serum and urine. A BioTek Synergy H1 96-well microplate reader (Winooski, VT, USA) was used to measure absorbance and/or fluorescence. Determinations were carried out in duplicate samples and normalized to 1 mg of total protein. Total protein content was determined colorimetrically using the bicinchoninic acid (BCA) method (Thermo Scientific PIERCE BCA Protein Assay (Rockford, IL, USA)). Bovine serum albumin (BSA) was used as a standard.

### 2.6. Protein Glycoxidation Products

The content of KN, NFK, DT and TRY were assessed fluorometrically, measuring fluorescence at 365/480, 325/434, 330/415 and 295/340 nm, respectively. Following this, urine and serum samples were diluted in 0.1 M H_2_SO_4_ (1:5, *v*/*v*) directly before determination. Later, the results were normalized to the fluorescence of 0.1 mg/mL quinine sulfate (in 0.1 M H_2_SO_4_) and expressed in arbitrary fluorescence units (AFU)/mg protein [41].

### 2.7. Antioxidant Assays

Cu-Zn superoxide dismutase (SOD) activity in serum and urine was assessed spectrophotometrically at 480 nm, measuring the rate of inhibition of epinephrine oxidation. One unit of SOD activity was defined as the amount of enzyme that inhibits 50% of the epinephrine oxidation [42]. 

Serum and urine catalase (CAT) activity was determined spectrophotometrically at 240 nm by assessing the distribution of hydrogen peroxide (H_2_O_2_). One unit of CAT activity was described as the amount of enzyme catalyzing the breakdown of 1 mM H_2_O_2_ per min [43]. 

Serum glutathione peroxidase (GPx) activity was assessed spectrophotometrically at 340 nm, based on the reduction reaction of organic peroxides by GPx in the presence of decreased nicotinamide adenine dinucleotide phosphate (NADPH) [44].

Reduced glutathione (GSH) concentration was measured by a colorimetric method developed by Ellman using 5,5-dithio-bis-(2-nitrobenzoic acid) DTNB, whereas the absorbance was measured at 412 nm [45].

### 2.8. Oxidative and Nitrosative Stress Markers

The end product of oxidation 4-Hydroxynonenal (4-HNE) was determined using a colorimetric lipid peroxidation assay. For that purpose, a methasulfonic-acid-based medium at 586 nm was used [46]. 

The levels of total nitric oxide (NO), peroxynitrite and S-Nitrosothiols in serum and urine were measured spectrophotometrically at 490, 320 and 490 nm, respectively. Total NO was determined using sulfanilamide and N-(1-naphthyl)-ethylenediamine dihydrochloride (NEDA⋅2 HCl). Peroxynitrite concentration in serum and urine was assessed by peroxynitrite-mediated nitration, resulting in the formation of nitrophenol. The level of S-nitrosothiols in serum and urine was determined by the reaction of Griess reagent with Cu^2+^ ions [47,48].

### 2.9. Statistical Analysis

The data were presented as a number of cases with a certain percentage for the qualitative data and a median with an interquartile range for the quantitative data. The normality of data distribution was assessed using the Shapiro–Wilk test. Differences in quantitative variables between groups were assessed using the Mann–Whitney test for variables with non-normal distribution and the *t*-test for data with normal distribution. The Wilcoxon–Mann–Whitney test was used in paired comparisons. The differences in qualitative variable distributions were assessed using the Chi-square test. The Spearman’s rank correlation coefficient was employed to evaluate the correlations. The analyses were performed using the R programming language in the RStudio environment (R 3.3.0+). The *p* values < 0.05 were considered significant for the study.

## 3. Results

### 3.1. Demographics

The study and control groups were compatible in terms of age, gender, relationship status and BMI, but the depressed subjects were more prone to be smokers and had lower education levels. The study group consisted of 33 patients with depression, including 21 with a positive history of COVID-19. After the antidepressant treatment of 4–6 weeks, a reassessment was performed on 21 patients, 15 of whom had a history of COVID-19. Twelve patients did not undergo the second examination due to various reasons (faster discharge, lack of cooperation or contact, discontinuation of the study). The control group consisted of 30 patients, 23 of whom had been infected with COVID-19 in the past (Table 2). Due to the small number of smokers in control group (*n* = 3), we conducted a statistical analysis only for the patients. The application of the Mann–Whitney test did not reveal significant differences between smoking and non-smoking patients in O&NS and kynurenine pathway parameters, CRP and D-dimers.

### 3.2. Comparison between Study and Control Groups

#### 3.2.1. O&NS and Kynurenine Pathway Parameters, CRP and D-Dimers

Significantly increased concentration of TRY was observed in the study group before treatment compared to the control group. Moreover, a significant difference was observed in serum peroxynitrite, TRY and DT concentrations between the study group at the second measurement and the controls. Peroxynitrite concentrations were significantly lower, while DT and TRY were characterized by higher concentrations in the study group after undergoing the antidepressant treatment (Table 3). 

There were no significant differences in the study group between the concentrations of CRP and D-dimers in measurements before and after the treatment and the controls (Table 3). Furthermore, no significant differences were observed in relation to COVID-19 between the study group in the first measurement and the control group.

Additionally, in depressed patients with a history of COVID-19, we observed lower levels of GPx in serum (*p* = 0.008) and significantly higher levels of NO in urine (*p* = 0.033) in the first measurement. Meanwhile in the second measurement, higher levels of S-Nitrosothiols (*p* = 0.045) in serum were found. In the control group, significantly higher levels of NFK in urine were determined in COVID-19 survivors (*p* = 0.048) (Table 4).

#### 3.2.2. Scales Assessing Severity of Depression and Anxiety, Results of Cognitive Tests and Stress Related to the COVID-19 Pandemic

In the study group, the scores of scales assessing the severity of depression and anxiety were significantly higher before and after the antidepressant treatment compared to the control group. No significant differences were observed between the pre-treatment depressed patients and the control group in IES-R scores (Table 2), also in relation to COVID-19 history. Significantly lower scores in tests of cognitive functions (VFT, TMT Parts A&B, DST, SCWT and CVLT) were observed in the depressed patients before treatment than in the control group. However, after undergoing the antidepressant treatment, significantly lower scores in depressed patients were limited to the Stroop Color Word Test (Table 5).

### 3.3. Comparison of the Results of the First and Second (before and after Antidepressant Treatment) Measurements in the Study Group

#### 3.3.1. O&NS and Kynurenine Pathway Parameters, CRP, and D-Dimers

A significant decrease in serum peroxynitrite levels (*p* < 0.000) and an elevation of GSH levels (*p* = 0.046) were observed in serum after the antidepressant treatment. Other than that, no significant differences were observed. No statistically significant differences in inflammatory parameters CRP and D-dimers were detected (Table 6).

#### 3.3.2. Scales Assessing Severity of Depression and Anxiety and Results of Cognitive Tests

After the treatment, there was a significant decrease in the severity of depression and anxiety assessed by HAM-D (*p* = 0), BDI (*p* = 0) and HAM-A (*p* = 0), and an increase in the scores of individual CVLT tasks (Trial 1, Trials 1–5, List B, Short-Delay Free Recall, Short-Delay Cued Recall, Long-Delay Free Recall) assessing memory processes (Table 7).

### 3.4. Correlations of Oxidative Stress, CRP and D-Dimers with Individual Parameters

In the study group, there was no significant correlation of the concentrations of oxidative stress parameters from the first measurement to be performed alternating in HAM-D, HAM-A and BDI scores after the treatment. We observed a positive correlation of CRP values with a reduction of depression severity assessed by BDI after the antidepressant treatment (r = 0.5; *p* = 0.0169).

In the depressed patients before the treatment, we observed positive correlations of HAM-D scale scores with serum CAT and urinary S-Nitrosothiols levels, as well as positive correlations of BDI scores with serum GSH and SOD levels. In addition, there was a positive correlation of IES-R scores with serum GSH levels before treatment in the study group (Table 8).

### 3.5. Correlations of SARS-CoV-2 Antibodies with Individual Parameters

In the study group before and after antidepressant treatment, no significant correlations were found between the levels of SARS-CoV-2 antibodies and the severity of depression and anxiety, the change in the scores of the HAM-D, HAM-A, BDI scales and the severity of general symptoms during SARS-CoV-2 infection. 

On the other hand, a significant correlation was observed in the control group between the levels of anti-N IgG antibodies and the severity of taste disorders during SARS-CoV-2 infection (r = 0.44; *p* = 0.0182). The observed correlation was positive, meaning that those with higher levels of antibodies had more severe symptoms during their COVID-19 infection.

## 4. Discussion

A growing number of studies point to the relevance of inflammation in the pathomechanism of the development of depression, which is accompanied by an increase in O&NS causing impaired brain function and modulation of neurotransmission [49,50]. Depression is associated with altered levels of oxidative stress markers and impaired total antioxidant status. This includes usually decreased concentrations of certain antioxidant compounds, such as glutathione (GSH), or enzymes, including glutathione peroxidase (GPx), catalase (CAT) and superoxide dismutase (SOD) [51,52]. However, some sources report either an increase or no change in the concentration of antioxidant enzymes [53,54,55,56]. A study by Bilici et al. indicates that increased severity of depression is characterized by significantly higher levels of certain antioxidant enzymes, including erythrocyte SOD and GPx [49]. Other studies confirm the linkage between oxidative stress and depression as well as the significant positive correlation between disease severity and SOD activity [50,57]. The results of this study indicate that O&NS and an increase in antioxidant enzymes are associated with the severity of depression. In the patients with depression before treatment, we observed positive correlations of HAM-D scale scores with serum CAT and urinary S-Nitrosothiols, as well as positive correlations of BDI scores with serum GSH and SOD. Increased levels of antioxidant enzymes may be the result of a compensatory effect [58]. During oxidative stress and inflammation, GSH synthesis is upregulated [59], which may explain our results of the correlation between the severity of the Beck Depression Inventory and higher GSH. S-Nitrosothiols as antioxidants protect against oxidative damage [60]. Therefore, their increase may also indicate the body’s response to stress. It is worth mentioning that, in this group of patients, we found a positive correlation between IES-R scores and serum GSH levels in the first measurement. Research indicates reduced GSH levels in depression and other conditions associated with oxidative stress [56,61,62,63]. However, increased levels of the main antioxidant GSH may indicate antioxidant protection against cell death [64,65], which may also appear in the case of severe emotional stress related to COVID-19. In addition, we observed higher TRY levels in the group of depressed patients before antidepressant treatment versus the control group. However, these data are not consistent with numerous studies showing reduced TRY levels in depressed patients. It is related to the excessive activation of the enzyme indole 2,3-dioxygenase (IDO) catabolizing tryptophan to kynurenine and its metabolites [17]. Nevertheless, a study presented by Nobis et al. showed similar results to the current study [66]. It can be only theorized that the increased levels of TRY in depressed patients may derive from the catabolic degradation of body proteins at the onset of depression/inflammation and TRY release. This leads to the conclusion that protein and TRY reserves may be depleted in the chronic stage of the disorder. Similarly, the activity of antioxidant enzymes may initially increase during the inflammatory phase, but their reserves may be depleted during depression.

Oxidative stress is associated with the neurodegenerative process characteristic of depression and cognitive decline [49,57,67]. Several studies prove the presence of deficits in the areas of memory, attention, executive functions and psychomotor speed in depressed patients compared to healthy individuals and their symmetrical correlation with the severity of depression and the number of episodes [68]. We confirmed reports of reduced cognitive functions in depressed individuals in comparison to healthy individuals, as evidenced by significantly lower scores in tests assessing cognitive functions (VFT, TMT Part A&B, DST, SCWT and CVLT). 

A meta-analysis by Osimo et al. found that more than half of depressed patients present slightly elevated CRP levels, and about a quarter of patients show signs of low-grade inflammation [69]. This indicates the impact of inflammation in the course of depression. In addition, psychological stress caused the symptoms of depression and anxiety and induced a chronic low-grade hypercoagulable state, which may be linked to elevated D-dimers in the aforementioned group of depressed patients [70]. The survival of COVID-19 may be associated with persistent increases in CRP and D-dimer levels, indicating long-lasting inflammation in the body, even up to several months after combating the virus. This has been demonstrated by a growing number of studies [71,72,73,74]. Therefore, one would expect that in patients with depression and after COVID-19, persistent inflammation would be expressed by higher CRP and D-dimer parameters. However, in our study, we did not confirm these hypotheses. We did not observe statistically significant differences in CRP and D-dimer parameters between the control group and the study group, in patients before and after the antidepressant treatment as well as their history of COVID-19.

A study by Saleh et al. showed continued oxidative stress in the brain with decreased gray matter glutathione (GSH) levels several months after infection in subjects with a history of COVID-19 [75]. In their study, Stufano et al. reported that oxidative damage persists in subjects with prior COVID-19 infection even four months after SARS-CoV-2 infection [76]. This suggests a possible role of oxidative stress mediators in the pathogenesis of long COVID, meaning long-term symptoms after the infection. In our study, we found higher urinary NO levels and lower serum GPx levels in depressed patients with a history of COVID-19 before the antidepressant treatment. Higher levels of nitrosative stress biomarkers and lower levels of GPx, which is involved in protecting cells from toxicity, may indicate a greater contribution of inflammation in patients with depression and a history of COVID-19. In healthy controls with a history of COVID-19, significantly higher levels of NFK, a biomarker of protein damage, were observed. 

In their study, Hampshire et al. supported the hypothesis that post-COVID-19 individuals (both hospitalized and non-hospitalized cases) may have permanent and significant cognitive deficits [77]. In addition, based on an analysis of a 2-year retrospective cohort study of individuals diagnosed with COVID-19, Taquet et al. found an increased risk of cognitive deficits, dementia and other neuropsychiatric disorders [78]. Moreover, in their study, Latronico et al. observed an improvement in cognitive functions over time from SARS-CoV-2 infection, while symptoms of depression, anxiety and post-traumatic stress disorder, present after 3 months, remained unchanged [79]. However, in this study, we did not confirm the hypothesis of a significant effect of COVID-19 infection on cognitive functions scores in healthy controls, and we did not find differences in subjects with depression before and after treatment in the context of COVID-19. No correlation was found between the level of anti-SARS-CoV-2 antibodies and the individual results of cognitive tests. This may be dependent on the number of studied subjects and the time passed since the illness, which also indicates a questionable effect of COVID-19 intercourse on cognitive functions in people with depression. 

So far, the results of studies demonstrating an increased risk of psychological distress after COVID-19 are mixed, due to evidence of mitigating the effects of infection over time [80]. However, a large study analyzing data from over 50,000 participants found an association between COVID-19 exposure and later mental distress, depression, anxiety and overall lower life satisfaction, showing no evidence for a link between COVID-19 and gender, education and pre-pandemic mental health [81]. Even so, our study did not support this hypothesis since there was no greater severity of depression and anxiety found in both the study group and the control group due to the COVID-19 illness. There was also no correlation found between the results in the HAM-D, HAM-A and BDI scales and levels of antibodies to SARS-CoV-2. Although some studies indicate that the level of SARS-CoV-2 antibodies depends on the severity of COVID-19 infection [82], in our study among study participants, we did not observe a correlation of SARS-CoV-2 levels with the severity of general symptoms during SARS-CoV-2 infection. However, we observed a significant correlation between the level of anti-N IgG antibodies indicating past COVID-19 and the severity of taste disorders during SARS-CoV-2 infection. This may indicate that a stronger immune response leads to more pronounced taste disorder symptoms. In their study, Kwasniewska et al. verified that taste and olfactory symptoms in younger patients correlated with lower antibodies levels [83]. These results are not consistent with our evidence but may be due to the consideration of combining taste and olfactory disorders as opposed to our considerations.

Antidepressant treatment has immunomodulatory properties. It can normalize oxidative stress parameters and increase the activity of some neuroprotective antioxidant enzymes [51,84]. However, there are studies indicating an ambiguous effect of antidepressant treatment on the modulation of oxidative stress. In the brain, antioxidant properties were most frequently demonstrated, but in the liver and testicular cells, most studies showed pro-oxidant effects. Studies show that effective antidepressant treatment reduces inflammation, and higher inflammation inhibits the response to antidepressants [85]. In a meta-analysis by Gasparini et al., patients who did not respond to antidepressants had higher baseline levels of C-reactive protein and interleukin-8, which indicated an abnormal inflammatory process [86]. Our study does not support this hypothesis, as we observed an association between higher CRP values and an improvement in BDI scores after the antidepressant treatment. However, after the inclusion of the antidepressant treatment (lasting 4–6 weeks), significantly decreased levels of peroxynitrite, a byproduct of NO synthesis and a key oxidant in redox processes in pathological conditions, were observed in patients in the study group. In addition, there were significantly increased serum levels of GSH, which is the most important peroxynitrite scavenging antioxidant. Moreover, higher levels of DT, a marker of oxidative protein damage, were observed after the antidepressant treatment, differently to the control group. This may cast doubt on the exclusively antioxidant effects of this group of drugs. However, it could also be due to a small study group or too short duration of the treatment, as Sarandol et al. indicate that 6-week antidepressant treatment has no effect on oxidative systems [50]. In addition, significant reductions in depression and anxiety severity and improvements in cognitive functions (CVLT tasks -Trials 1–5, Trial 1, List B, Short-Delay Free Recall, Short-Delay Cued Recall, Long-Delay Free Recall) were observed in depressed patients after antidepressant treatment. So far, several studies have confirmed the positive effect on cognitive functions after antidepressant treatment [87,88,89,90]. 

In the context of COVID-19 history, no association was observed between the level of SARS-CoV-2 antibodies and the response to antidepressant treatment expressed by changes in the HAM-D, HAM-A and BDI scales. Moreover, in the second measurement, depressed patients with a history of COVID-19 had higher serum levels of S-Nitrosothiols, which may indicate that the limited effect of the antidepressant therapy in these patients, due to the initial higher inflammation, is limited.

## 5. Conclusions

In our study, in line with existing knowledge, we confirmed that depression is closely related to increased inflammation, including O&NS, and is accompanied by a cognitive decline. We noted the correlation of depression severity with oxidative stress (CAT, GSH and SOD in serum, S-Nitrosothiols in urine). We also indicated that an effective antidepressant treatment has a modulating effect on oxidative stress parameters, clinical improvement of depressive symptoms and cognitive function scores. Even though we did not confirm the hypothesis that COVID-19 history could affect the clinical response to the antidepressant treatment in depressed patients, we did observe reduced levels of the antioxidant enzyme GPx in biochemical parameters and elevated levels of NO in urine, indicating increased oxidative stress. Moreover, in subjects with depression and a history of COVID-19, significantly higher serum levels of S-Nitrosothiols were noted in the second measurement, which may point to a limited biochemical response to antidepressant treatment. Perhaps in additional studies, an association could be discovered between the level of SARS-CoV-2 antibodies and the response to treatment as expressed by changes in depression and anxiety scales in depressed individuals. Studies indicate that the higher the inflammation, the weaker the response to antidepressant treatment, which can be a source of treatment ineffectiveness and resistance. 

Therefore, it is important to search for new therapeutic solutions and potentiation of antidepressant treatments in patients in the context of a history of SARS-CoV-2 infection. In the era of the COVID-19 pandemic and its consequences, it is important to conduct further research in this area, especially since depression is a potentially life-threatening disease.

## 6. Limitations

The limitations of this study include the small group size in the pre- and post-treatment antidepressant measurements as well as the heterogeneity of the groups in terms of education, smoking history and antidepressant treatment used during the study. Due to the different pharmacodynamic profiles, antidepressants may affect the parameters evaluated in our study differently. Moreover, the study does not include a comparison of depressed patients on antidepressant treatment with depressed patients without treatment, which is a limitation of the study. Furthermore, due to the hindered cooperation with depressed patients or technical difficulties, some data are incomplete.

## Figures and Tables

**Table 1:** The antidepressant treatment used during the study.

Antidepressant Treatment	*n*	%	Maximum Dose (mg)
Escitalopram	1	3.0	10
Fluoxetine	1	3.0	40
Sertraline	3	9.1	100
Duloxetine	7	21.2	90
Venlafaxine	3	9.1	225
Amitriptyline	1	3.0	150
Vortioxetine	1	3.0	10
Mirtazapine	1	3.0	45
Duloxetine + bupropion	3	9.1	60 + 150

**Table 2:** Demographic and clinical characteristics of all subjects.

	Controls (*n* = 30)	MDD Patients (*n* = 33)	*p*-Value	Treatment Response Evaluation (*n* = 21)
Demographic Information				
Age (median, Q1–Q3)	42 (34.5–54)	44 (26–55)	0.746	43 (21–50)
BMI (median, Q1–Q3)	23.8 (21.7–26.4)	24.7 (21.2–27.5)	0.449	24.2 (20.8–26.9)
Sex (% female)	66.60%	60.60%	0.8126	61.90%
Education (%)				
6–9 years	0 (0.0)	4 (12.1)	<0.001	2 (9.5)
10–14 years	7 (23.3)	25 (75.8)		16 (76.2)
>15 years	23 (76.7)	4 (12.1)		3 (14.3)
Relationship status (%)				
Single	8 (26.7)	16 (48.4)	0.126	13 (61.9)
In a relationship	22 (73.3)	17 (51.5)		8 (38.1)
Smoking status (%smokers)	3 (10)	15 (45.4)	0.003	8 (38.1)
Clinical Information				
COVID-19 confirmed history (%)	23 (76.6)	21(63.6)	0.395	15 (71.4)
Severity of symptoms	4 (2.25–5.78)	2 (0–5)	0.248	2 (0–5)
Taste disorders (%)	15 (50)	8 (24.2)	0.1894	6 (28.5)
Smell disorders (%)	16 (53.3)	7 (21.2)	0.4005	6 (28.5)
Episodes (%)				
1	0	6 (18.1)	<0.0001	6 (28.5)
2 or more	0	27 (81.8)		15 (71.5)
Hamilton Depression Rating Scale (HAM-D) median (Q1–Q3)	0 (0–0)	22 (18–27)	<0.001	5 (3–10)
Beck Depression Inventory (BDI) median (Q1–Q3)	4 (1–6.75)	29 (21–41)	<0.001	11 (6–22)
Hamilton Anxiety Rating Scale (HAM-A) median (Q1–Q3)	0 (0–0)	22 (14–26)	<0.001	4 (1.5–9.5)
Impact of Event Scale—Revised (IES-R) median (Q1–Q3)	19.5 (7–30.2)	22.5 (12.2–48.5)	0.2156	23 (14–18)

**Table 3:** O&NS and kynurenine pathway parameters, CRP, D-dimers in study and control groups.

Serum/Urine	Variable	Control Group (*n* = 30)			Pre-Treatment Group (*n* = 33)			*p* *	Post-Treatment Group (*n* = 21)			*p* **
		Median	Q1	Q3	Median	Q1	Q3		Median	Q1	Q3	
Serum	CAT, nmol H_2_O_2_/min/mg protein	1.55	1.26	1.74	1.39	1.06	1.76	0.561	1.54	1.43	1.72	0.563
	DT, AFU/mg protein	11.1	8.05	12.5	11.8	10.3	13.6	0.095	12.6	10.6	14.6	0.02
	GSH, μg/mg protein	1.02	0.894	1.13	0.892	0.818	1.05	0.054	1.09	0.932	1.14	0.438
	4-HNE, umol/mg protein	6.21	5.7	6.43	6.22	5.82	6.55	0.77	5.94	5.06	6.47	0.447
	KN, AFU/mg protein	15	9.23	18.1	16.3	11.5	18.9	0.299	16	14.5	17.8	0.306
	NFK, AFU/mg protein	8.6	5.51	11.8	10.8	7.95	11.9	0.149	9.43	8.68	11.7	0.283
	NO, nmol/mg protein	5.37	2.2	15.7	7.19	1.13	19.6	0.453	10.4	2.95	23.3	0.547
	Peroxynitrite, nmol/mg protein	30.1	18.8	40.5	24.7	16.7	41.6	0.522	12.9	11.9	15.1	0.000
	GPx, mU/mg protein	1.43	1.34	1.55	1.49	1.4	1.55	0.189	1.47	1.38	1.5	0.572
	S-Nitrosothiols, nmol/mg protein	3.51	3.12	3.93	3.54	3.28	3.94	0.373	3.47	3.11	3.75	0.915
	SOD, mL/mg protein	0.287	0.211	0.359	0.288	0.22	0.349	0.778	0.193	0.041	0.365	0.106
	TRY, AFU/mg protein	137	108	158	160	145	170	0.008	158	139	171	0.026
Urine	CAT, nmol/H_2_O_2_/min/mg protein	0.881	0.746	1.27	0.94	0.766	1.46	0.416	0.975	0.851	1.22	0.337
	DT, AFU/mg protein	48.2	29.7	65.6	50.9	34.8	76.4	0.69	57.1	40.8	77.2	0.302
	GSH, μg/mg protein	1.21	0.914	1.6	1.27	1.13	1.84	0.215	1.44	1.16	1.72	0.059
	4-HNE, umol/mg protein	5.02	3.79	7.04	5.31	3.69	8.88	0.79	5.36	4.17	7.17	0.711
	KN, AFU/mg protein	37	30.7	47.4	43.1	33.9	53	0.431	46.4	35.8	61.6	0.073
	NFK, AFU/mg protein	17.4	11.4	24.7	18.3	12.2	29.7	0.647	22.6	15.3	32.5	0.179
	NO, nmol/mg protein	2.64	1.42	3.96	2.38	1.07	3.21	0.516	2.39	1.27	3.59	0.628
	Peroxynitrite, nmol/mg protein	7.49	5.73	9.06	7.46	6.32	11.8	0.431	7.47	6.64	9.96	0.827
	GPx, mU/mg protein	1.37	0.973	1.96	1.51	0.988	2.38	0.599	1.4	1.18	2.1	0.628
	S-Nitrosothiols, nmol/mg protein	4.84	3.98	6.34	5.68	4.75	6.81	0.072	5.57	4.94	6.25	0.049
	SOD, mL/mg protein	0.119	0.074	0.253	0.209	0.098	0.338	0.205	0.225	0.134	0.401	0.051
	TRY, AFU/mg protein	5.81	4.01	8.41	6.63	4.93	12	0.247	7.04	5.17	12.7	0.124
Serum	CRP, mg/L	0.8	0.525	1.45	0.6	0.3	1.7	0.313	0.75	0.4	1.25	0.626
	D-dimers, ng/mL	401	349	509	456	387	530	0.072	457	338	524	0.558

*p* *—control group vs. pre-treatment group; *p* **—control group vs. post-treatment group. Abbreviations: CAT, catalase; DT, dityrosine; GSH, reduced glutathione; 4-HNE, 4-hydroxynonenal; KN, kynurenine; NFK, N-formylkynurenine; NO, nitric oxide; GPx, glutathione peroxidase; SOD, superoxide dismutase; TRY, tryptophan; CRP, C-reactive protein

**Table 4:** O&NS and kynurenine pathway parameters of participants with and without COVID-19 history.

Variable	Non-COVID			COVID			*p*
	Median	Q1	Q3	Median	Q1	Q3	
	First measurement (COVID *n* = 21, non-COVID *n* = 12)						
NO urine, nmol/mg protein	1.33	0.93	2.15	3.06	1.77	4.96	0.033
GPx serum, mU/mg protein	1.54	1.51	1.64	1.46	1.39	1.51	0.008
	Second measurement (COVID *n* = 15, non-COVID *n* = 6)						
S-Nitrosothiols serum, nmol/mg protein	3.13	2.86	3.42	3.66	3.21	3.92	0.045
	Control group (COVID *n* = 23, non-COVID *n* = 7)						
NFK urine, AFU/mg protein	13.4	11.3	14	20.6	12.5	27.8	0.048

Abbreviations: NO, nitric oxide; GPx, glutathione peroxidase; NFK, N-formylkynurenine.

**Table 5:** Results of cognitive tests and clinical scales in study and control groups.

Variable	Control Group (*n* = 30)			Pre-Treatment Group (*n* = 33)			*p* *	Post-Treatment Group (*n* = 21)			*p* **
	Median	Q1	Q3	Median	Q1	Q3		Median	Q1	Q3	
VFT Animals Category	24.5	21	29	20	17	23.2	0.012	20	16	24.5	0.116
VFT Letter “K”	18	17	20	13	8	18	0.001	17	12	20.5	0.284
VFT Letter “S”	14	11.2	19	10	8.5	13	0.002	12	10	14.5	0.085
Digital Span Task WAIS-R	11	10.2	15	10	8	12.5	0.027	11	9	15.5	0.451
TMT A	20	17.2	25.5	25.5	2.5	36.2	0.001	21	17.5	33	0.271
TMT B	42	38.2	56.8	65	51.8	94.5	0.001	56	41	86	0.121
SCWT word-reading	21	19	22.8	24.5	22.8	27	0	23	22	27	0.004
SCWT color-naming	50	43	55.8	59	50	80.2	0.006	58	50	64.5	0.051
Trials 1–5	7	5	8	5	3	6	0.004	7	6	8	0.312
Trial 1	6	4.25	7	4	3	5	0.001	7	6	8	0.145
List B	6	5	7.75	6	4	7	0.508	6	5	8.5	0.461
Short-Delay Free Recall	7	6	9	5.5	4	7	0.009	7	5.5	8	0.654
Short-Delay Cued Recall	8	6.25	10	4.5	4	7.25	0.003	7	5.5	10	0.565
Long-Delay Free Recall	8	5.25	10	5	4	8	0.01	8	6	8.5	0.867
Long-Delay Cued Recall	7	5.25	10	5	4	6	0	6	5.5	9	0.636
HAM-D	0	0	0	22	18	27	0	5	3	10	0
BDI	4	1	6.75	29	21	41	0	13	6.25	22.8	0
HAM-A	0	0	0	22	14	26	0	4	1.5	9.5	0

*p* *—control group vs. pre-treatment group; *p* **—control group vs. post-treatment group. Abbreviations: VFT, the Verbal Fluency Test; TMT, Trail Making Test; SCWT, Stroop Color Word Test; HAM-D, the Hamilton Depression Rating Scale; BDI, the Beck Depression Inventory; HAM-A, the Hamilton Anxiety Rating Scale.

**Table 6:** O&NS and kynurenine pathway parameters, CRP, D-dimers in study group: pre- and post-treatment.

Serum/Urine	Variable	Pre-Treatment Group (*n* = 21 *)			Post-Treatment Group (*n* = 21)			*p*
		Median	Q1	Q3	Median	Q1	Q3	
Serum	CAT, nmol H_2_O_2_/min/mg protein	1.39	1.12	1.76	1.54	1.43	1.72	0.473
	DT, AFU/mg protein	11.7	10.3	13.5	12.6	10.6	14.6	0.153
	GSH, μg/mg protein	0.892	0.818	1.05	1.09	0.932	1.14	0.046
	4-HNE, umol/mg protein	6.06	5.58	6.48	5.94	5.06	6.47	0.946
	KN, AFU/mg protein	17.1	11.1	19.1	16	14.5	17.8	0.869
	NFK, AFU/mg protein	10.8	7.95	11.8	9.43	8.68	11.7	0.661
	NO, nmol/mg protein	6.14	1.07	15.1	10.4	2.95	23.3	0.609
	Peroxynitrite, nmol/mg protein	36.8	16.6	56.8	12.9	11.9	15.1	0.000
	GPx, mU/mg protein	1.48	1.42	1.51	1.47	1.38	1.5	0.785
	S-Nitrosothiols, nmol/mg protein	3.55	3.07	3.93	3.47	3.11	3.75	0.681
	SOD, mL/mg protein	0.291	0.203	0.351	0.193	0.041	0.365	0.055
	TRY, AFU/mg protein	160	143	172	158	139	171	0.812
Urine	CAT, nmol H_2_O_2_/min/mg protein	1.01	0.75	1.59	0.975	0.851	1.22	0.901
	DT, AFU/mg protein	55.2	35.6	81.6	57.1	40.8	77.2	0.767
	GSH, μg/mg protein	1.27	1.13	1.84	1.44	1.16	1.72	0.452
	4-HNE, umol/mg protein	495	3.69	10.1	5.36	4.17	7.17	0.946
	KN, AFU/mg protein	44.2	34.4	53.4	46.4	35.8	61.6	0.338
	NFK, AFU/mg protein	19.2	12.3	33.1	22.6	15.3	32.5	0.412
	NO, nmol/mg protein	2.07	1.04	4.96	2.39	1.27	3.59	0.609
	Peroxynitrite, nmol/mg protein	7.46	6.21	12.1	7.47	6.64	9.96	0.973
	GPx, mU/mg protein	1.56	1.01	2.65	1.4	1.18	2.1	0.946
	S-Nitrosothiols, nmol/mg protein	5.68	4.72	7.17	5.57	4.94	6.25	0.892
	SOD, mL/mg protein	0.172	0.072	0.362	0.225	0.134	0.401	0.432
	TRY, AFU/mg protein	6.84	4.93	14.7	7.04	5.17	12.7	0.946
Serum	CRP, mg/L	0.5	0.3	1.7	0.8	0.4	1.3	0.943
	D-dimers, ng/mL	456	383	551	457	338	524	0.225

* Matched cases only. Abbreviations: CAT, catalase; DT, dityrosine; GSH, reduced glutathione; 4-HNE, 4-hydroxynonenal; KN, kynurenine; NFK, N-formylkynurenine; NO, nitric oxide; GPx, glutathione peroxidase; SOD, superoxide dismutase; TRY, tryptophan; CRP, C-reactive protein.

**Table 7:** Results of cognitive test and clinical scales in study group: pre- and post-treatment.

Variable	Pre-Treatment Group (*n* = 21 *)			Post-Treatment Group (*n* = 21)			*p*
	Median	Q1	Q3	Median	Q1	Q3	
VFT Animals Category	20	18	23	20	16	24.5	0.600
VFT Letter “K”	14	10	18	17	12	20.5	0.190
VFT Letter “S”	11	10	13,5	12	10	14.5	0.757
Digital Span Task WAIS-R	10	8	12,5	11	9	15.5	0.090
TMT A	24	21	34,5	21	17.5	33	0.111
TMT B	65	49	98	56	41	86	0.421
SCWT word-reading	23	22	27	23	22	27	0.473
SCWT color-naming	58	49.5	73.5	58	50	64.5	0.230
Trial 1	4	3	5	7	6	8	0
Trials 1–5	5	3	6	7	6	8	0.001
List B	6	4	7	6	5	8.5	0.026
Short-Delay Free Recall	5	4	7	7	5.5	8	0.015
Short-Delay Cued Recall	4	4	7	7	5.5	10	0.008
Long-Delay Free Recall	4	4	7.5	8	6	8.5	0.005
Long-Delay Cued Recall	5	4	6	6	5.5	9	0.001
HAM-D	20	19	27	5	3	10	0
BDI	28	21	43	11	6	22	0
HAM-A	22	14	26	4	1.5	9.5	0

* Matched cases only. Abbreviations: VFT, the Verbal Fluency Test; TMT, Trail Making Test; SCWT, Stroop Color Word Test; HAM-D, the Hamilton Depression Rating Scale; BDI, the Beck Depression Inventory; HAM-A, the Hamilton Anxiety Rating Scale.

**Table 8:** Statistically significant correlations between oxidative stress parameters and clinical variables in study group before treatment.

First Variable	Second Variable	r	*p*	Method
HAM-D	CAT serum, nmol H_2_O_2_/min/mg protein	0.38	0.0289	Spearman
HAM-D	S-Nitrosothiols urine, nmol/mg protein	0.38	0.027	Spearman
BDI	GSH serum, μg/mg protein	0.39	0.0251	Spearman
BDI	SOD serum, mI/mg protein	0.42	0.0154	Spearman
IES-R	GSH serum, μg/mg protein	0.52	0.00424	Spearman

Abbreviations: HAM-D, the Hamilton Depression Rating Scale; BDI, the Beck Depression Inventory; IES-R, the Impact of Event Scale—Revised; CAT, catalase; GSH, reduced glutathione; SOD, superoxide dismutase.

## Data Availability

Data are contained within the article.
